# A Rare Case of Allergic Bronchopulmonary Aspergillosis Progressing to Cardiac Tamponade in the Al Qassim Region of Saudi Arabia

**DOI:** 10.7759/cureus.40531

**Published:** 2023-06-16

**Authors:** Ahmad AlJarallah, Sami Alharbi, Seetah M Alharbi, Hessa A Alsaaf

**Affiliations:** 1 Internal Medicine and Pulmonology, King Fahad Specialist Hospital, Buraydah, SAU; 2 Internal Medicine, King Fahad Specialist Hospital, Buraydah, SAU

**Keywords:** ccp (cyclic citrullinated peptide), anti-double-stranded dna antibodies (anti-dsdna), antinuclear antibodies (ana), antineutrophil cytoplasmic antibodies (anca), basic metabolic panel (bmp), rheumatoid factor (rf), shortness of breath (sob), tuberculosis(tb), allergic bronchopulmonary aspergillosis(abpa)

## Abstract

Allergic bronchopulmonary aspergillosis (ABPA) is a condition characterized by an exaggerated response of the immune system (a hypersensitivity response) to the fungus *Aspergillus*. *Aspergillus*-associated pericarditis leading to pericardial tamponade is rare. In our case, we presented a case of a 22-year-old female asthmatic patient with no other medical conditions who presented to the emergency department (ED) complaining of severe chest tightness and shortness of breath. Echocardiography revealed significant pleural and pericardial effusion consistent with cardiac tamponade. Both pleural and pericardial fluids were hemorrhagic. Four months later, she presented to the ED with chief complaints of shortness of breath and a cough lasting two days. She was admitted as a case of asthma exacerbation. In the following months, when the patient visited the pulmonology outpatient clinic, the doctors recommended for specific IgE test. Allergen-specific IgE testing was positive for *A. fumigatus* to confirm the presence of ABPA. As we rolled out other causes of cardiac tamponade, we link the development of cardiac tamponade secondary to an underlying *Aspergillus* infection. We report this case with the aim of improving clinical knowledge regarding probable causes of cardiac tamponade in patients with asthma, which may facilitate the establishment of early diagnosis and treatment protocols.

## Introduction

Allergic bronchopulmonary aspergillosis (ABPA) is a condition characterized by an exaggerated response of the immune system (a hypersensitivity response) to the fungus *Aspergillus*. There is a high prevalence of this condition in individuals suffering from uncontrolled asthma, cystic fibrosis, and those with an immunocompromised status. Nearly all patients reported to have ABPA have also been clinically diagnosed with asthma [1]. ABPA typically presents as bronchospasm, pulmonary infiltration, and eosinophilia.

Patients often demonstrate detectible immunologic evidence of allergies to antigens of *Aspergillus* species [2].

Cardiac tamponade is a serious medical condition defined by an accumulation of fluid in the pericardial sac that compresses the heart, resulting in decreased venous return and shock. Cardiac tamponade is caused by a variety of factors, such as cancer, kidney failure, chest trauma, myocardial infarction, and pericarditis [3].

*Aspergillus*-associated pericarditis leading to pericardial tamponade is rare [4]. This paper describes the case of a patient with chronic asthma who developed cardiac tamponade, ultimately being diagnosed with ABPA.

## Case presentation

A 22-year-old female patient with asthma presented to the emergency department (ED) complaining of severe chest tightness and shortness of breath (SOB). The patient had no prior history of joint pain, oral or nasal ulcers, and no family history of asthma, respiratory illness, or cardiac disease. For her past history, the patient had been diagnosed with asthma three years prior for which she was taking (budesonide/formoterol) and (tiotropium bromide) inhalers. On examination, she looked ill and dyspneic with a raised jugular venous pressure. The following vital signs were recorded: blood pressure (BP) was 105/66; heart rate (HR) was 135 beats/min; respiratory rate (RR) was 25 breaths/min; and SPO_2_ was maintained at 95% on a 15 L nonrebreather mask. The patient was admitted to the cardiac intensive care unit (ICU) for further treatment. 

**Figure 1:**
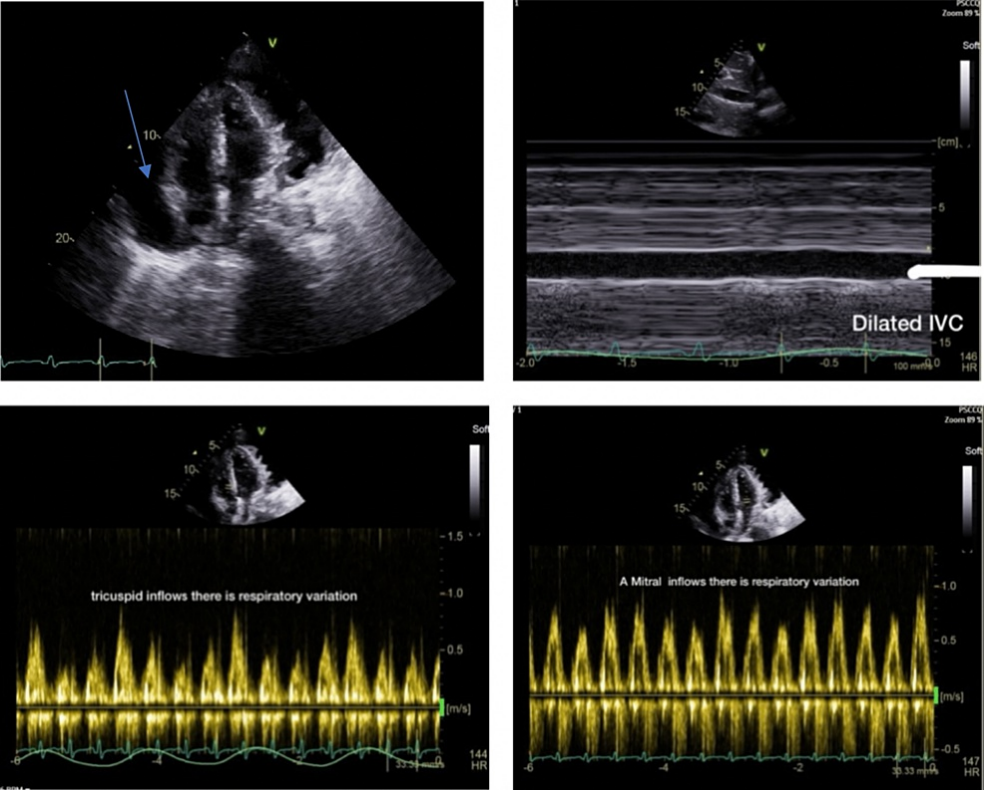
Echocardiography It revealed significant pericardial effusion (blue arrow), and also it showed dilated inferior vena cava (IVC) with respiratory changes in mitral and tricuspid inflows consistent with cardiac tamponade.

Echocardiography revealed significant pleural and pericardial effusion consistent with cardiac tamponade (Figure 1).

She underwent pericardiocentesis and thoracentesis during admission, requiring aspiration of 500 mL pericardial fluid and 350 mL pleural fluid. Fluid analysis, including a Gram stain, fluid culture, and a polymerase chain reaction-based test for tuberculosis (TB) and Coxsackie virus, was negative; nevertheless, both pleural and pericardial fluids were hemorrhagic, where the count of white blood cells (WBCs) in the pericardial fluid was 200 cells/microliter (normal: <500 cells/microliter) with 72% neutrophils, 22% lymphocytes, 4% monocytes, and 2% eosinophils, and the red blood cells (RBCs) were positive (+3). Regarding the chemistry of the pericardial fluid, the creatinine level was 22.0 µmol/L, the protein level was 32.4 g/L, the amylase level was 5.0 U/L, the lactate dehydrogenase (LDH) level was 177 U/L, and albumin level was 16.8 g/L. 

Complete blood count (CBC ) showed WBC count = 11 × 10^3^/uL with eosinophils count being 1.7 × 10^3^/uL. 

**Figure 2:**
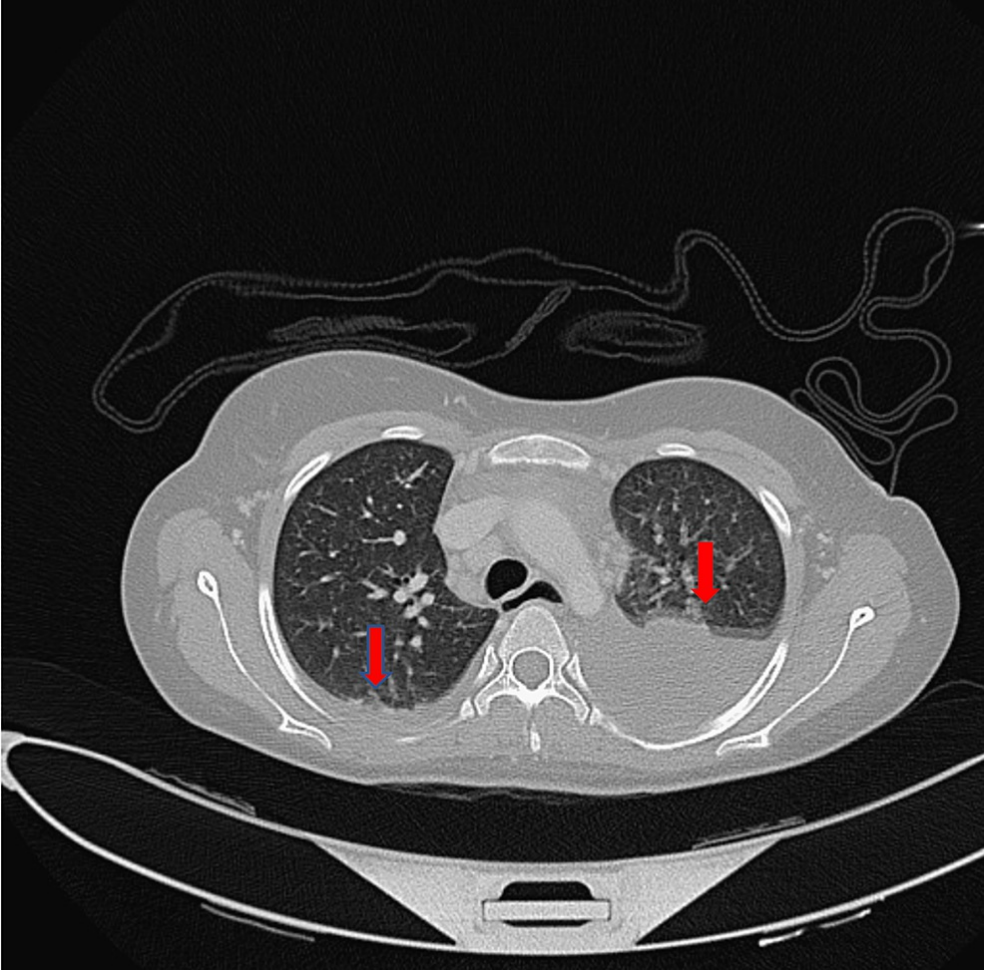
Chest CT It showed bilateral pleural effusion (red arrows) associated with bilateral lower lobe lung collapse. Pericardial effusion was also observed. CT, computed tomography.

The patient was also negative for *Brucella* based on a serology test. Also, negative connective tissue disease workup included antinuclear antibody (ANA), antineutrophil cytoplasmic antibodies (ANCA), anti-double-stranded deoxyribonucleic acid (anti-DsDNA) antibody, and anti-cyclic citrullinated peptide (anti-CCP) findings. Rheumatoid factor (RF) levels were normal. Blood cultures showed no growth. Chest computed tomography (CT) showed moderate bilateral pleural effusion associated with bilateral lower lobe lung collapse (Figure 2). Pericardial effusion was also observed.

The patient was discharged after she started improving and maintained oxygen at 96% on room air. She was discharged with oral colchicine 0.5 mg, twice daily, with follow-up at cardiology and pulmonology clinics. Four months later, she presented to the ED with chief complaints of SOB and cough lasting two days. The cough was productive with greenish sputum. Her SOB worsened during night. She also experienced several episodes of post-tussive vomiting that contained food particles and no blood. She was hospitalized at our facility due to severe asthma exacerbation and community-acquired pneumonia. She was administered nebulized Atrovent 500 mcg every 8 h, nebulized Ventolin 2.5 mg every 6 h, methylprednisone 40 mg intravenous two times daily, ceftriaxone 1 g intravenous daily, and oral azithromycin 500 mg daily.

A physical examination performed on hospital admission revealed bilateral basilar crackles with scattered bronchi. All other findings were normal. All labs, including a PCR-based COVID-19 test, ANCA, ANA, Ds-DNA, LDH, D-dimer, and basic metabolic panel (BMP), were normal except an eosinophil count of 2 × 10^3^ cells/uL and the total IgE of 3418 g/L being elevated, raising suspicion of ABPA. Unfortunately, allergen-specific IgE testing was unavailable at that time in our hospital.

**Figure 3:**
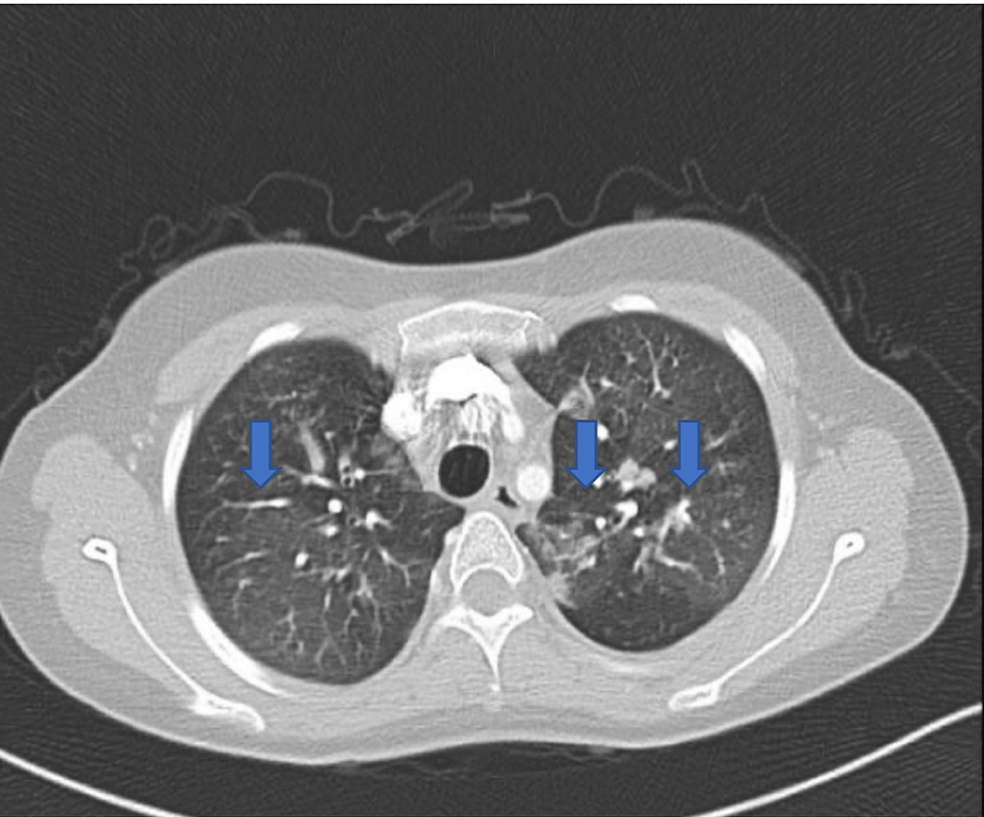
Chest CT It showed multiple scattered ground-glass opacities (blue arrows), and no filling defect was observed in the pulmonary artery or its branches, ruling out the possibility of pulmonary embolism. CT, computed tomography.

Chest CT revealed multiple scattered ground-glass opacities indicative of an infective process; however, no filling defect was observed in the pulmonary artery or its branches, ruling out the possibility of pulmonary embolism (Figure 3).

The patient was discharged after seven days. She improved clinically and maintained oxygen at room air. She was given prednisone for five days and budesonide/formoterol inhalers with follow-up at a pulmonology clinic. 

After one month, the patient visited the pulmonology outpatient department (OPD), and she showed slight improvement. Workup of severe asthma was performed, revealing the following: negative anti-CCP and ANCA, normal RF, ERS of 30, eosinophil count of 2 × 10^3^ cells/uL, total IgE of 3418 g/L, IgG of 15 g/L (7.38-16.21), and IgM of 1.52 g/L (0.511-1.95). A sputum culture produced no growth. Allergen-specific IgE testing was positive for *A. fumigatus* to confirm the presence of ABPA.

**Figure 4:**
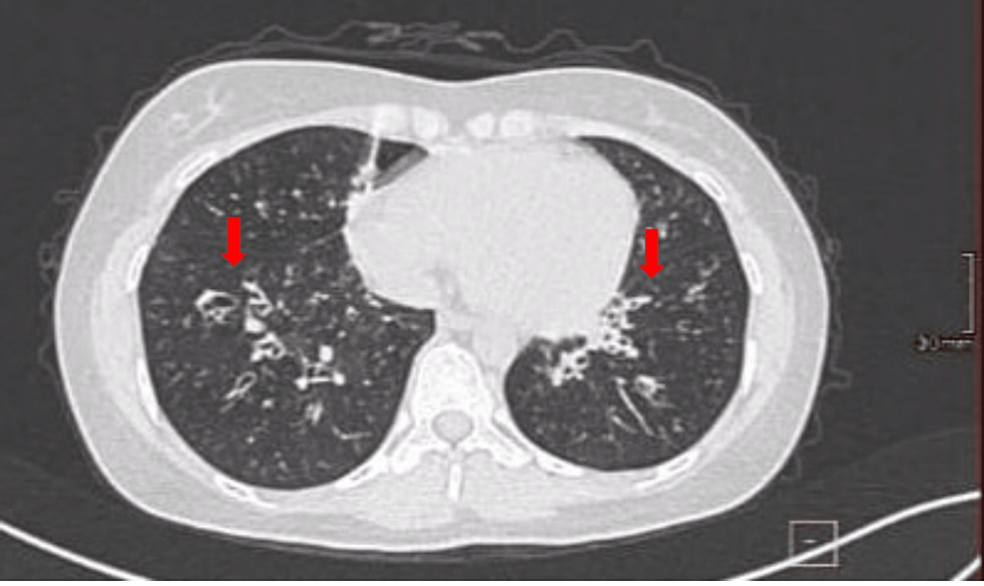
Chest CT image A chest CT image revealing central bronchiectasis (red arrows). CT, computed tomography.

Chest CT revealed multiple scattered ground-glass opacities with central bronchiectasis (Figure 4). 

Based on a diagnosis of ABPA, an initial dosage of 0.5 mg/kg prednisolone therapy was initiated, which was tapered over a period of three months. The patient was followed up every eight weeks, and her condition dramatically improved.

## Discussion

Cardiac tamponade due to aspergillosis-induced pericarditis is incredibly challenging to diagnose, particularly among patients without a prior diagnosis of pulmonary aspergillosis. In our patient, a diagnosis of ABPA was established three months after the patient was admitted and treated for cardiac tamponade. The most common presenting complaints and signs of patients with *Aspergillus* pericarditis are chest pain/tightness, cardiac tamponade, and pericardial friction [5]. A study of 28 cases of *Aspergillus* pericarditis, conducted by Le Moing et al., found that the condition mainly occurs in individuals with severely weakened immune systems and it is usually caused by the spread of *Aspergillus* from the lung or heart muscle. Tamponade was found in 28% of the cases of aspergillosis [6].

Patients with a predisposition to aspergillosis often have underlying medical conditions, with cancer being the most common at 44%. Other prevalent conditions include bone marrow transplantation (25%), solid-organ transplantation (13%), HIV/AIDS (3.8%), autoimmune disease (2%), and systemic steroid use (3.5%) [5]. In this particular case, the patient had been diagnosed with asthma three years prior and had experienced exacerbations, possibly due to undiagnosed ABPA.

The symptoms of aspergillosis affecting the heart can vary but the most common form of infection is myocardial aspergillosis, which accounts for up to 83% of cases. It often occurs along with endocarditis or pericarditis [5]. Even though the patient is often asymptomatic, the disease can present as an abnormal heart rhythm, which is usually diagnosed by an autopsy [7].

**Figure 5:**
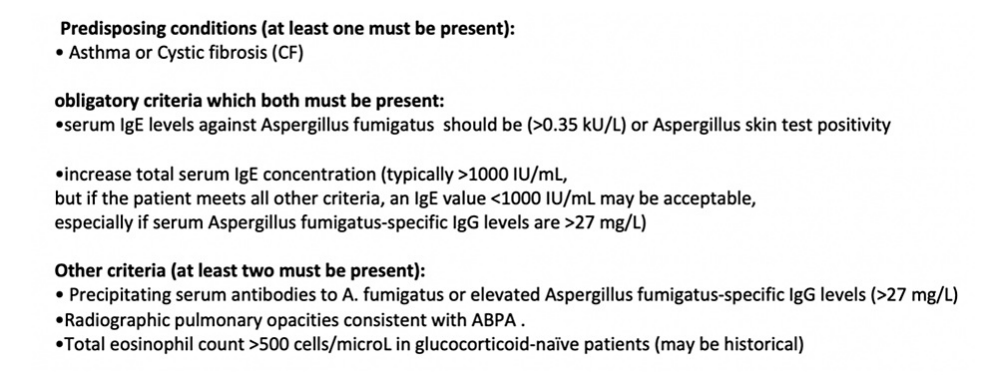
Diagnostic criteria for ABPA ABPA, allergic bronchopulmonary aspergillosis.

For the diagnosis of ABPA, we have diagnostic criteria proposed by the International Society for Human and Animal Mycology for ABPA (Figure 5) [8,9]. Our patient matched the criteria, that is, she had asthma. Also, her lab showed positive serum IgE levels against *Aspergillus fumigatus* with total serum IgE concentration >1000 IU/mL and total eosinophil count >500 cells/microL.

Pulmonary *Aspergillus* overlap syndromes have been reported earlier in many case reports. It may progress from one entity to another. The risk factors for *Aspergillus* overlap syndromes include genetic factors, the presence of severe underlying lung disease, and corticosteroid therapy [10].

Concurrent aspergilloma and ABPA are subsequent worsening of the disease with more frequent exacerbations of asthma [11].

Invasive pulmonary aspergillosis has been reported in patients with ABPA. According to some reports, *Aspergillus* species invade the tissues surrounding the bronchiectatic segments and are associated with granulomatous reactions [12].

Systemic glucocorticoids are the mainstay of treatment in acute ABPA. In a series of experiments conducted with 126 patients who were diagnosed with ABPA, they developed remission within six weeks after the course of oral glucocorticoids [13].

Furthermore, the Infectious Diseases Society of America guidelines on the treatment of aspergillosis recommended that the treatment of acute or recurrent ABPA must include a combination of itraconazole and glucocorticoids [14]. As in our case, she improved after a course of glucocorticoids.

## Conclusions

This report illustrates a rare case of cardiac tamponade in a patient diagnosed with ABPA. The diagnosis of cardiac tamponade resulting from pericardial aspergillosis is particularly challenging due to the lack of characteristic signs and symptoms. Despite its rarity, this condition can be fatal, particularly in individuals with compromised immunity. Therefore, it is imperative that clinicians maintain a high degree of clinical suspicion in order to facilitate early detection and diagnosis. This is of particular importance in patients who present with both pericardial effusion and asthma, as they tend to exhibit a poor response to treatment.
